# In-depth characterization of accessory gene regulator loci and associated virulence factors in *tcdA^+^B^+^ Clostridioides difficile* isolates

**DOI:** 10.1016/j.crmicr.2025.100435

**Published:** 2025-07-01

**Authors:** Mansoor Kodori, Zohreh Ghalavand, Abbas Yadegar, Gita Eslami, Masoumeh Azimirad, Mohammad Reza Zali

**Affiliations:** a Noncommunicable Diseases Research Center, Bam University of Medical Sciences, Bam, Iran; b Foodborne and Waterborne Diseases Research Center, Research Institute for Gastroenterology and Liver Diseases, Shahid Beheshti University of Medical Sciences, Tehran, Iran; c Department of Microbiology, School of Medicine, Shahid Beheshti University of Medical Sciences, Tehran, Iran; d Gastroenterology and Liver Diseases Research Center, Research Institute for Gastroenterology and Liver Diseases, Shahid Beheshti University of Medical Sciences, Tehran, Iran

**Keywords:** Clostridioides difficile, Accessory gene regulator (agr) system, Quorum sensing, Virulence determinants, Sporulation efficiency

## Abstract

*Clostridioides difficile* infection (CDI), along with the severity of the disease, is intimately tied to the production of bacterial toxins. The expression of toxins is governed by intricate mechanisms that respond to various intra- and intercellular stimuli. Recent genomic studies have identified an accessory gene regulator (*agr*) system in *C. difficile*, similar to that found in *Staphylococcus aureus*, playing a pivotal role in coordinating toxin synthesis. Nevertheless, the *agr* system of *tcdA^+^B^+^ C. difficile* clinical isolates, particularly in terms of phylogenetic analysis, phenotypic characteristics, and virulence expression, has not been extensively studied in Iranian isolates. This investigation aimed to characterize *agr* type and *agrD* sequences in *tcdA^+^B^+^* clinical isolates obtained from Iranian diarrheal patients over a two-year period, utilizing specifically designed primer sets. Basal expression levels of virulence and regulatory factors, genotyping, sporulation efficiency, and motility were also examined. PCR analysis of 50 *tcdA^+^B^+^ C. difficile* isolates revealed universal presence of *agr1*, with 44 (88 %) isolates also possessing *agr2*. A notable number of isolates (*n* = 12, 24 %) exhibited a threonine (T) to lysine (K) substitution at position 47 (T47K) in the AgrD1 C-terminal charged region, predominantly associated with RT126/*tcdC-A*/toxinotype V genotype. The AgrD2 sequence remained conserved across 44 isolates, aligning with R20291 strain. The study uncovered significant variability in sporulation efficiency among isolates, with high efficiency correlating with *tcdC-A* genotype. Furthermore, 70 % of isolates demonstrated motility, with 58 % showing high motility in semi-solid brain heart infusion broth medium. Our findings highlight widespread prevalence of *agr1* and the previously unrecognized T47K substitution within *agr1*, along with the distribution of two *agr* loci in *tcdA^+^B^+^ C. difficile* isolates. This study also provides novel phenotypic insights into *tcdA^+^B^+^ C. difficile* isolates from Iranian patients, paving the way for more comprehensive functional and clinical studies of *agr* loci.

## Highlights

• The study uncovers the universal presence of *agr1* and the notable T47K mutation in *tcdA^+^B^+^ Clostridioides difficile* isolates, highlighting significant genetic diversity.
• 87 % *tcdA^+^B^+^ C. difficile* isolates possess the *agr2* locus, emphasizing its potential role in the pathogen's lifecycle and virulence.
• Significant variability in sporulation efficiency and motility among *tcdA^+^B^+^* isolates is observed, with high sporulation efficiency strongly correlated with the *tcdC-A* genotype.

## 1 Introduction

*Clostridioides difficile*, formerly known as *Clostridium difficile*, is the leading cause of nosocomial antibiotic-associated diarrhea (AAD) and pseudomembranous colitis (PMC), conditions associated with significant morbidity and mortality (Smits et al., 2016). The pathogenicity of *C. difficile* is primarily due to its ability to produce two major exotoxins, TcdA and TcdB, along with other virulence factors that facilitate immune evasion and host cell colonization during *C. difficile* infection (CDI) (Chandrasekaran and Lacy, 2017; Awad et al., 2014). The global prevalence of CDI has increased over the past decade, potentially due to the emergence of hypervirulent or epidemic strains (Bauer et al., 2017). Pathogenic bacteria, including *C. difficile*, utilize gene regulatory networks to adapt rapidly to various host cell conditions during infection. These networks, in response to host immunological challenges, influence the physiological, metabolic, and virulence traits of the pathogen (Papenfort and Vogel, 2010). Two-component systems (TCSs) play a crucial role in linking external and internal signals to gene expression (Chakravarty and Massé, 2019). In *C. difficile*, approximately 10 % of the genome consists of TCS transcriptional regulators and signaling molecules, though their roles in virulence and pathogenesis remain to be fully elucidated (Stabler et al., 2009; Sebaihia et al., 2006).

Several regulatory systems in *C. difficile* have been identified, such as transcriptional regulator autoinducer 2 (AI-2), catabolite control protein A (CcpA), CodY protein, and RNA polymerase sigma-H factor (SigH). These are thought to influence the expression of *tcdA, tcdB*, and *Spo0A* genes. The flagellar regulon is also implicated in host cell adherence, colonization, and *in vitro* regulation of toxin production (Martin-Verstraete, Peltier, and Dupuy, 2016; Slater et al., 2019; Lee and Song, 2005; Antunes et al., 2012). Genome analyses of epidemic *C. difficile* strains have uncovered a genetic element resembling the accessory gene regulator (*agr*) locus of *Staphylococcus aureus* (Stabler et al., 2009; Sebaihia et al., 2006; Knight et al., 2017). The *agr* quorum-sensing locus (*agrACDB*) serves as a global regulator of virulence gene expression in *S. aureus*, responding to environmental stimuli (Choudhary et al., 2018). The *agrD* gene encodes a small, secreted cyclic autoinducing peptide (AIP), while the *agrB* gene encodes a transmembrane protein responsible for processing and exporting AIP. Accumulation of extracellular AIP activates AgrC, a sensor kinase, leading to phosphorylation of the AgrA response regulator. Activated AgrA binds to DNA, initiating the transcription of RNAII and the divergent RNAIII transcripts (Choudhary et al., 2018; Tan et al., 2018). *C. difficile* strains can possess *agr* loci in two configurations: *agr1*, which includes an incomplete *agr*-like locus with *agrD1B1* and appears to be found in all clinical strains, and *agr2*, which comprises the full *agr* operon (*agrA2C2D2B2*) (Darkoh et al., 2015; Darkoh, Odo, and DuPont, 2016; Martin et al., 2013). The *agr* operon in *S. aureus* categorizes strains into four types (*agr* types I through IV) based on mutations in the AgrC sensor domain and polymorphisms in AgrD peptides (Choudhary et al., 2018; Tan et al., 2018). While few studies have explored the *agr* loci in *C. difficile* isolates, no data is available on *agr* loci in isolates from Iranian CDI patients (Darkoh, Odo, and DuPont, 2016; Okada et al., 2020). This study aims to characterize the *agr* loci and virulence factors in a collection of *tcdA^+^B^+^ C. difficile* isolates with known genotypes [ribotype (RT), toxinotype, and *tcdC* genotype] from diarrheal patients in Tehran, Iran. Additionally, sporulation efficiency and motility of all isolates were assessed.

## 2 Materials and methods

### 2.1 Ethics statement

This study was conducted at the Department of Anaerobic Bacteriology, in the Research Institute for Gastroenterology and Liver Diseases, Tehran, Iran. Ethical approval was obtained from the Ethical Review Committee of Shahid Beheshti University of Medical Sciences (Project No.IR.SBMU.MSP.REC.1398.736). Numerical identifiers were assigned to each patient to maintain confidentiality of clinical and demographic data.

### 2.2 Isolates and DNA extraction

The study included 50 clinical *C. difficile* isolates positive for *tcdA* and *tcdB* genes, collected over a two-year period from diarrheal patients. Patient demographic information, antibiotic and medication history and clinical data were recorded for all subjects. Isolates were revived from storage by culturing on cycloserine-cefoxitin-fructose agar (CCFA, Mast) supplemented with 7 % horse blood at 37 °C for 48–72 h under anaerobic conditions (85 % N_2_, 10 % CO_2_, and 5 % H_2_) using the Anoxomat® Gas Exchange System (Mart Microbiology BV), as described in previous studies (Azimirad et al., 2020; Kodori et al., 2020). Pure colonies were suspended in 1 ml of molecular biology-grade water, and DNA was extracted using the InstaGene Matrix kit (Bio-Rad, USA) following the manufacturer's protocol. DNA purity and concentration were measured with a NanoDrop® ND-1000 spectrophotometer (Thermo Scientific). DNA samples were stored at −20 °C for subsequent use.

### 2.3 Detection of toxins genes

Two multiplex PCR formats were employed to detect *tcdA, tcdB, cdtA, cdtB*, 16S rRNA, *tcdE, tcdR, cdu2*, and *cdd3* genes, as previously described (Persson, Torpdahl, and KJCm, 2008; Cohen, Tang, and Silva, 2000). **Supplementary Table S1** lists the oligonucleotide primers and product sizes for each target gene.

### 2.4 Genotyping

Genotyping data for all isolates were included from a prior study (Kodori et al., 2020). In summary, amplification of *tcdC* gene using C1 and C2 primers was performed to determine *tcdC* genotype of isolates (Spigaglia and Mastrantonio, 2002). Sequencing was conducted on an ABI 3130XL Genetic Analyzer (Applied Biosystems, Foster City, *CA*, USA), and raw sequences were processed using Chromas Lite v2.5.1 and BioEdit v7.2.5 software. All genotypes were submitted to the NCBI database and deposited in GenBank. Additionally, toxinotyping of isolates was performed using a PCR-RFLP method, and capillary electrophoresis (CE) PCR ribotyping was conducted at the Department of Medical Microbiology, Motol University Hospital, Prague, Czech Republic, employing a consensus PCR ribotyping technique (Rupnik et al., 1998; Fawley et al., 2015). CE ribotyping profiles were compared to the WEBRIBO database (Indra et al., 2008).

### 2.5 Determinants genes and *agr* loci primer design

Complete and available *C. difficile agr* loci (*agr1* and *agr2*), as well as *codY, fbp68, fliD*, and *luxS* nucleotide sequences, were retrieved from the NCBI GenBank database. Specific primers were designed using CLC Sequence Viewer 8 based on conserved regions of these genes across different *C. difficile* strains. The primers were developed from the nucleotide sequences of *agr* and determinant genes (*codY, fbp68, fliD*, and *luxS*) of *C. difficile* strain R20291 (accession number: CP029423.1). The designed primers were aligned to all *C. difficile* strains using BLAST (BLAST, 2012).

### 2.6 PCR for *agr* loci and determinates genes

The presence of *agr* genes and determinant genes (*codY, slpA, fliD, fbp68, cwp84*, and *luxS*) was detected via PCR in a total volume of 25 μl, consisting of 12 μl 2 × master mix (Amplicon), 1 µl (10 pmol) of each primer, 2 μl (10 ng/µl) template DNA, and 9 μl distilled water. Primer sets and product sizes for each PCR are listed in **Supplementary Table S2**.

### 2.7 *agrD1* and *agrD2* sequence analysis

The *agrD1* and *agrD2* genes were amplified by PCR and purified using the Silica Bead DNA Gel Extraction Kit (Thermo Scientific, Fermentas, USA) in a 25 µl reaction volume. Purified PCR products were sequenced on both strands using an ABI 3730XL sequencer (Macrogen, Seoul, Korea). Chromas Lite v2.5.1 and BioEdit v7.2.534 were used for sequence editing. Nucleotide and amino acid sequences were aligned to the *agrD1* and *agrD2* sequences of the *C. difficile* reference strain R20291. Sequence analysis for SNPs and amino acid substitutions was conducted using BioEdit software v7.2.5. Complete nucleotide sequences of *agrD1* and *agrD2* were deposited in the NCBI GenBank database.

### 2.8 RNA extraction, cDNA synthesis, and real-time PCR

*C. difficile* isolates were cultured on CCFA agar (BD Biosciences) supplemented with 7 % horse blood and incubated at 37 °C for 48–72 h under anaerobic conditions. Single colonies were subcultured to saturation in brain heart infusion broth (BHI) and grown anaerobically. Liquid cultures (1 ml) were centrifuged at 2000 × *g* for 15 min, washed with PBS, resuspended in fresh BHI at a 1:50 ratio, and grown anaerobically. Optical density (OD600) was measured hourly. RNA was extracted from mid-exponential (OD600 = 0.5) and stationary growth phase cells using the RiboExTM RNA purification kit (GeneAll Biotechnology Co., Ltd.), measured with a NanoDrop® ND-1000 spectrophotometer, and treated with DNase (DNA freeTM Kit, Thermo Fisher). Reverse transcription was performed using the BioFACT cDNA kit, and relative basal expression levels were determined via real-time PCR on a Rotor-Gene® Q system (Qiagen). The 2^−ΔCT^ method calculated the ratio of target transcript expression relative to *rpoA* housekeeping gene. Reactions were performed in biological triplicates, with experiments conducted three times. Primer details for real-time PCR are listed in **Supplementary Table S2**.

### 2.9 Sporulation efficiency

Sporulation rates were measured using an alcohol shock sporulation assay (Camorlinga et al., 2019). Fresh 48-hour anaerobic cultures on Brucella broth (Merck) were adjusted to 0.5 McFarland, combined with 80 % ethanol, and incubated for 45 min. Serial dilutions were plated on Cassman Blood Agar (Merck). Colony formation was quantified after 72 h at 37 °C. Sporulation efficiency was calculated as:

$$\text{Sporulation}\;\text{efficiency}\;\left(\%\right)=\frac{\text{spore}\;}{\text{Vegetative}\;\text{cells}+\text{spore}\;}\times 100$$

### 2.10 Motility assay

Motility was assessed using a semi-solid medium as described by Valiente et al. (2012). Isolates were grown on BHI agar at 37 °C for 24–48 h anaerobically. BHI broth with 0.05 % agar was poured into 30 ml glass vials under anaerobic conditions. Three single colonies were inoculated onto the 0.05 % agar BHI medium and incubated anaerobically at 37 °C overnight. The maximum stalactite length was measured, and isolates were categorized as non-motile (<1 cm), motile (1–2 cm), or highly motile (>2 cm).

### 2.11 Phylogenetic analysis

Phylogenetic trees were constructed based on the deduced amino acid sequences of *tcdC* using Molecular Evolutionary Genetics Analysis version X (MEGA X) (Kumar et al., 2018). Sequence alignment was performed with ClustalW to infer genetic diversity and evolutionary relationships among *tcdC* sequences. A Maximum Likelihood phylogenetic tree was generated using the Poisson correction model for amino acid substitutions.

### 2.12 Statistical analysis

Statistical analyses were conducted using IBM SPSS Statistics v21 (IBM Corp.). The Chi-square test and Pearson correlation coefficient were used to identify possible associations. Statistical significance was defined as a *P*-value < 0.05.

## 3 Results

### 3.1 Isolates characteristics and genotypes

**Fig. 1:**
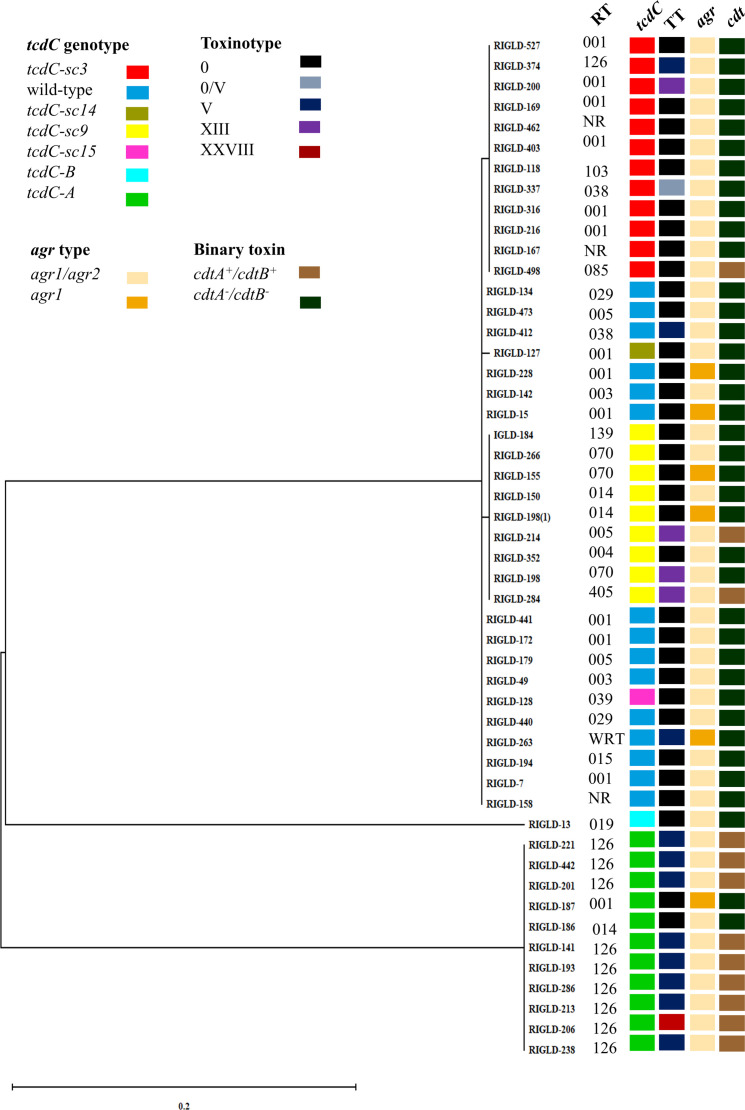
Phylogenetic analysis of the *tcdC* variants based on inferred amino acid sequences from 50 *C. difficile* isolates. A Maximum Likelihood phylogenetic tree was constructed using the Poisson substitution model in MEGA X, with bootstrap resampling at 1000 replications. The ribotype, toxinotype, *agr* profile, binary toxin presence, and *tcdC* genotype classifications are displayed for all isolates. RT: Ribotype; TT: Toxinotype; *cdt*: Binary toxin*.*

Among 50 *tcdA^+^B^+^* isolates confirmed as *C. difficile* through the presence of *cdu-2, cdd-3*, and 16S rRNA genes via PCR, all were positive for *tcdR* and *tcdE* genes within the pathogenicity locus (PaLoc). **Supplementary Table S3** presents demographic and clinical data of 50 CDI patients included in this work. CDT gene detection revealed that 24 % of the isolates concurrently harbored both *cdtA* and *cdtB* genes. The most frequently reported hospitalization ward was gastroenterology (38 %), with 78 % of the patients having a history of antibiotic use. The *tcdC* variant genotype was identified in 70 % of the isolates, while the remaining 30 % possessed the wild-type (non-variant) *tcdC* genotype. Additionally, 24 % and 18 % of the isolates corresponded to RT/toxinotype 001/0 and RT/toxinotype 126/V, respectively (Fig. 1). The most prevalent genotypes among the *tcdA^+^B^+^* isolates were toxinotype 0/*tcdC* wild type, toxinotype 0/*tcdC-sc3*, and toxinotype V/*tcdC-A* (**Supplementary Table S4**) (Kodori et al., 2020).

### 3.2 Prevalence of *agr* genes and virulence determinants

A conventional PCR was applied to detect *agr* genes in the *agr1* (*agrD1B1*) and *agr2* (*agrA2B2C2D2*) loci of *tcdA^+^B^+^* isolates (**Supplementary Table S2**). The results demonstrated that 44 (88 %) isolates harbored both *agr* loci, while 6 (12 %) isolates possessed only the *agr1* locus and lacked the *agr2* locus. Furthermore, all 50 isolates were found to contain *codY, fbp68, luxS, fliD, slpA, cwp84, spo0A*, and *ccpA* genes

### 3.3 *agrD1* and *agrD2* sequence analysis

**Fig. 2:**
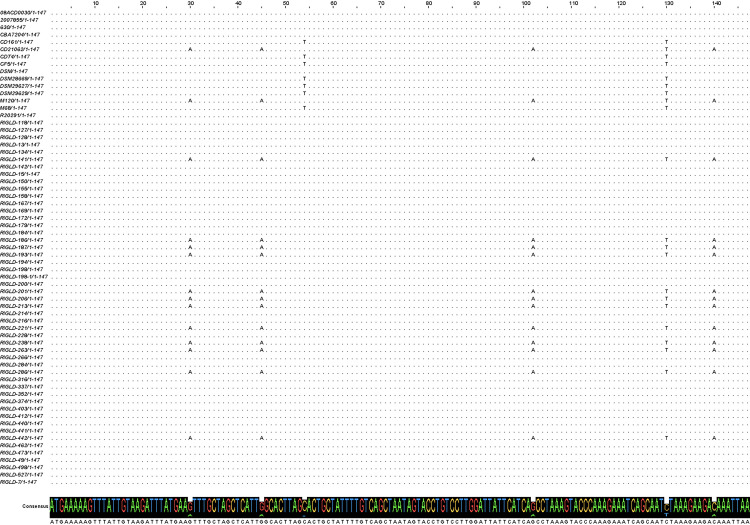
Sequence alignment of *agrD1* across 50 isolates and 15 reference strains. Nucleotide sequence alignment of *agrD1* across all isolates, and 15 reference strains retrieved from GenBank including R20291, CD630, M68, M120, CF5, DSM1296, 2007,855, CD21062, 08ACD0030, DSM29629, CD161, DSM29627, DSM28669, CBA7204, CDT4. The alignment was performed using Jalview software, to indicate SNPs and sequence variations.

**Fig. 3:**
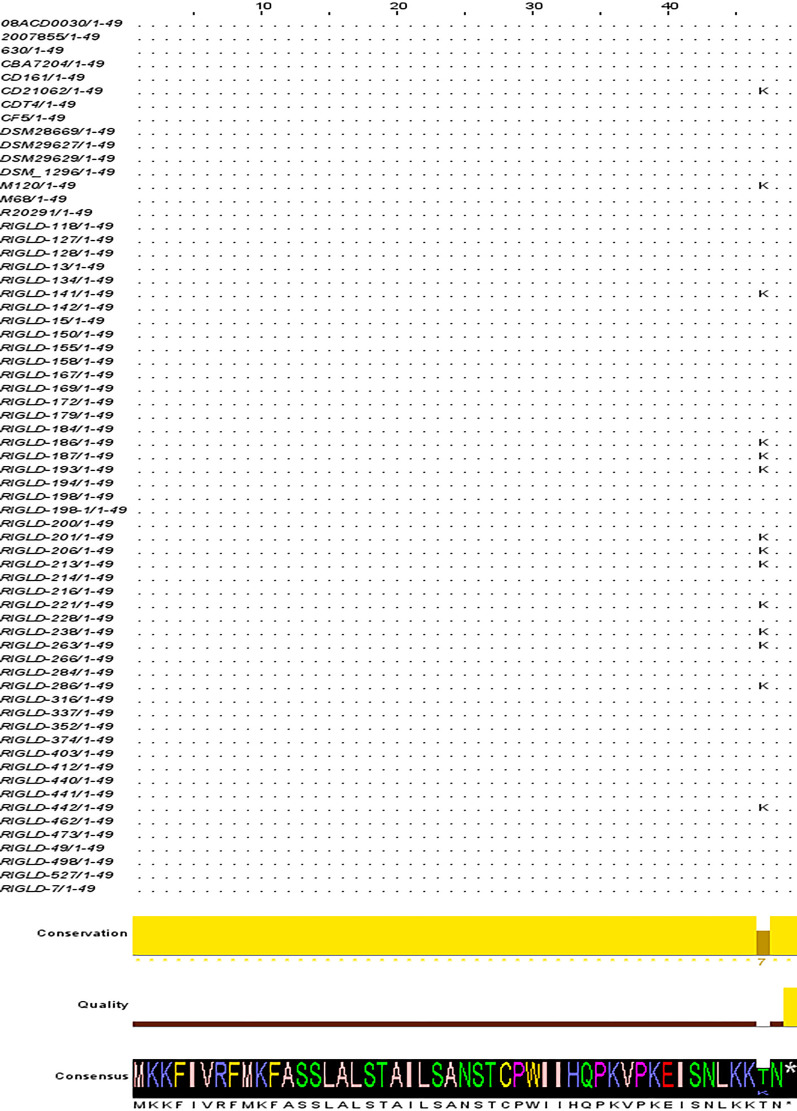
Amino acid sequence alignment of *agrD1* across all isolates and 15 reference strains retrieved from GenBank including R20291, CD630, M68, M120, CF5, DSM1296, 2007,855, CD21062, 08ACD0030, DSM29629, CD161, DSM29627, DSM28669, CBA7204, CDT4, showcasing conserved regions and mutations.

All isolates were investigated for the presence of *agrD1* (359 bp PCR product) and *agrD2* (297 bp PCR product) genes using PCR sequencing. Raw sequence data were manually edited and trimmed before comparison with sequences in the GenBank repository. The complete coding sequences (CDS) of *agrD1* and *agrD2* were found to be 147 bp (48 amino acids) and 141 bp (48 and 46 amino acids), respectively. Twelve isolates (24 %) exhibited multiple single nucleotide polymorphisms (SNPs) in their *agrD1* sequences compared to the R20291 and CD630 strain. These SNPs included G30A, G45A, G102A, C130T, and C140A substitutions. The C140A substitution resulted in a threonine (T) to lysine (K) mutation at position 47 of AgrD1 C-terminal charged region (Fig. 2). Moreover, comparative analysis of publicly available whole-genome sequencing (WGS) datasets from additional *C. difficile* strains (**Supplementary Table S5**) revealed the presence of this amino acid substitution within the genomic architecture of strains M120 and CD21062 (Fig. 3). Among the identified SNPs, all were synonymous mutations, resulting in no alteration of the encoded amino acid sequence. The twelve isolates classified within the polymorphism group exhibited 96.6–100 % sequence identity with *C. difficile* reference strains, including M120, as documented in the NCBI GenBank database. In contrast, the remaining 38 isolates, phylogenetically aligned with *C. difficile* strains R20291 and CD630, demonstrated 97.2–100 % sequence identity with publicly available GenBank sequences. Notably, nine isolates (75 %) of the polymorphism group possessed the RT126 genotype, with a significant correlation between the nucleotide substitutions and the RT126 genotype (*P*-value= 0.004). Additionally, 11 (91.6 %) and nine (75 %) isolates from this group exhibited the *tcdC-A* genotype, with significant correlations observed between the *tcdC-A* genotype and toxinotype V in terms of *agrD1* polymorphism (*P*-value= 0.004 for both). Sequence analysis of 44 clinical isolates of *C. difficile* revealed that all isolates had identical *agrD2* sequences to R20291 strain. Comparative sequence alignment showed that *agrD2* sequence shared 97.87–100 % homology with sequences available in the NCBI GenBank database.

### 3.4 Sporulation and motility capability

The ability of *C. difficile* isolates to sporulate, a key mode of transmission, was assessed using the agar plate method with alcohol shock treatment. Visible colonies grown over 48 to 72 h of anaerobic incubation showed sporulation efficiency ranging from 0.02 % to 18.9 %. Notably, 25 (50 %) of the clinical *tcdA^+^B^+^* isolates exhibited sporulation efficiency levels above 1 %. A significant correlation was found between sporulation efficiency and *tcdC* genotype, with the *tcdC-A* genotype demonstrating a higher sporulation rate (*P-*value= 0.048). Motility, essential for pathogen adhesion and colonization of the intestinal epithelium during CDI, was evaluated using a motility assay. Fresh colonies were stab-inoculated into the top of 0.05 % agar BHI vials. Based on movement in the semi-solid medium, *C. difficile* isolates were categorized as non-motile, motile, or highly motile. In this study, 70 % of all isolates exhibited motility in semi-solid BHI. Of these motile isolates, 58 % displayed high motility, defined as movement exceeding two centimeters.

### 3.5 Association between *agrD1* and *agrD2* gene expression and phenotypic characteristics

To quantify the basal expression levels of target genes relative to the housekeeping *rpoA* gene, total RNA was extracted from bacterial cells cultured during both mid-exponential and stationary growth phases **(Supplementary Figs. S1-S4)**. The fold change in expression for each target gene in relation to *rpoA* gene was calculated and analyzed for potential statistical correlations, as presented in Table 1.

**Table 1:** The gene expression level of *agr* genes and virulence determinants and their statistical correlations with phenotypic characteristics.

Target gene	Percent of isolates that had expression level more than *rpoA* gene	The observed positive correlation with other variables
*spo0A*	32	Sporulation efficiency (r_s_=0.325, *P*-value=0.021)
*ccpA*	46	*fliD* (r_p_=0.407, *P*-value=0.009)
*fbp68*	44*	–
*slpA*	38*	*spo0A* (r_p_=0.635, *P*-value=0.001); *tcdB* (r_p_=0.716, *P*-value=0.001); *fliD* (r_p_=0.893, *P*-value=0.001); *cwp84* (r_p_:0.330, *P*-value*=*0.019)
*codY*	34	*spo0A* (r_p_=0.657, *P*-value =0.001); *fliD* (r_p_=0.574, *P*-value =0.001); *cwp84* (r_p_=0.458, *P*-value =0.001); *luxS* (r_p_=0.402, *P*-value =0.004)
*luxS*	48^§^	*agrD1* (r_p_=0.938, *P*-value=0.001); *cwp84* (r_p_=0.938, *P*-value=0.001); *codY* (r_p_=0.938, *P*-value=0.001).
*cwp84*	46^§^	*fliD* (r_p_=321, *P*-value=0.023); *luxS (*r_p_= 0.938, *P*-value=0.0001); *codY* (r_p_:0458, *P*-value=0.001); *spo0A* (r_p_=0.363, *P*-value=0.01); *agrD1* (r_p_=408, *P*-value=0.003)
*fliD*	50	*ccpA* (r_p_=0.407, *P*-value=0.009); *spo0A* (r_p_=0.688, *P*-value=0001); *codY* (r_p_=0.574, *P*-value=0001); *slpA* (r_p_=0.983, *P*-value=0001); *tcdB* (r_p_=0.670, *P*-value=0.001)
*tcdA*	62*	*–*
*tcdB*	32€	*spo0A* (r_p_=0.442, *P*-value=0.001); *fliD* (r_p_=0.67 0, *P*-value=0.001).
*tcdC*	32€	*tcdR* (r_p_=0.966, *P*-value=0.0001)
*tcdR*	42€	*tcdC* (r_p_=0.966, *P*-value=0.0001)
*agrD1*	34*	*tcdA* (r_p_=0.58, *P*-value=0.001); *codY* (r_p_=0.321, *P*-value=0.023); *tcdC* (r_p_=0.519, *P*-value=0.001); *tcdR* (r_p_=0.544, *P*-value=0.001); *luxS* (r_p_=0.443, *P*-value=0.001); *cwp84* (r_p_=0.4408, *P*-value=0.003)
*agrD2*	25*	*–*

*>0.01 expression level of *rpoA*; § >0.1 expression level of *rpoA*

€ >0.001 expression level of *rpoA*; r_s_: Spearman's correlation coefficient, r_p_: Pearson correlation coefficient.

## 4 Discussion

*C. difficile* is the predominant cause of AAD and hospital-associated diarrhea worldwide, leading to significant morbidity and mortality, especially among hospitalized elderly patients (Czepiel et al., 2019). The primary virulence factors of *C. difficile* are toxins A and B, which are directly linked to the severity of CDI. However, the precise mechanisms governing toxin production and release into the host intestinal tract remain poorly understood. Recent studies suggest that toxin expression is intricately connected to regulatory networks controlling sporulation, motility, and key metabolic pathways (Majumdar and RJCoim, 2022). It is well established that quorum sensing modulates bacterial pathogenicity, and *agr* loci are crucial genetic factors in this regulation (Dotto et al., 2021). The *agr* system and its orthologues are present in the Firmicutes phylum, including *S. aureus*, and are associated with the expression of virulence and colonization factors in response to environmental stimuli (Wuster and MMJJob, 2008). Comparative array hybridization analysis of clinical *C. difficile* strains from Europe reveals that a significant portion of their genome consists of regulatory genes, accounting for 11 % of the genome content of CD630 strain. The CD630 strain contains only *agrD1* and *agrB1* genes (*agr1* locus), whereas the R20291 strain possesses an additional complete *agrC2A2B2D2* locus (*agr2*) (Marsden et al., 2010). Despite the known presence of the *agr* system in *C. difficile*, there has been a lack of studies on its regulatory role in clinical isolates from Iranian patients. This research is the first to examine the *agr* loci, toxin expression, and phenotypic characteristics of *tcdA^+^B^+^ C. difficile* clinical isolates from Iran.

In our study, we found incomplete *agr1* locus (*agrB1D1* genes) in all isolates, indicating the universality of *agr1* in *tcdA^+^B^+^* clinical isolates of *C. difficile*. Additionally, 88 % of the isolates contained a second complete *agr2* locus (*agrC2A2B2D2*) alongside the *agrB1D1* genes, while 12 % lacked the *agr2* locus. Furthermore, *codY, fbp68, luxS, fliD, slpA, cwp84, spo0A*, and *ccpA* genes were present in all *tcdA^+^B^+^* isolates. Marsden *et al*. were the first to report *agr* genes in a collection of clinical *C. difficile* strains, most from the UK and The Netherlands. They found that the additional *agr2* locus, as identified in R20291, is present in 86 % of the studied strains, including non-toxigenic strains. They suggested that the absence of *agrC2A2B2D2* genes might be an exception rather than the rule. They also concluded that *agrC2A2B2D2* locus is not necessarily linked to increased virulence or the emergence of hypervirulent strains (Marsden et al., 2010). Their results demonstrate that commonly occurring and emergent PCR RT markers are variably present in other RTs. In the presence of AI, the *agr-1* locus cannot initiate a quorum-sensing regulatory response due to the absence of *agrAC* response regulator genes. Consequently, *agr-1* mutant strains are unable to synthesize toxins and fail to colonize the gut or establish infection in murine models (Darkoh et al., 2015). In our study, there were no significant differences in *tcdA* and *tcdB* expression between isolates with or without the *agr2* genes. However, a statistical significance relation was observed between *tcdB* and *spo0A*, and *tcdB* and *fliD* basal expression levels. Ahmed *et al*. noted that deleting the *agr1* locus slightly affects toxin production, although *AgrD1* peptide accumulation could boost toxin gene transcription in the absence of *agrB1.* Additionally, it was found that deletion of *agr1* locus, or specifically *agrD1* and *agrB1* genes, significantly impacts the transcription of sporulation-related genes, resulting in a loss of sporulation capability. The absence of both *agrD1* and *agrB1* genes also leads to reduced motility (Ahmed et al., 2020). Interestingly, deletion of *agrB1*/*agrD1* or *agrD1* alone did not markedly affect *tcdA* expression and had minimal impact on the expression of *tcdR* and *tcdB*. Further research indicated that inactivation of *agrA* diminished toxin production in the R20291 strain (RT027). AgrA may have an indirect role in modulating flagellar synthesis and the expression of genes associated with c-di-GMP metabolism (Martin et al., 2013). In our analysis, we observed a significant correlation between the basal expression of *agrD1* and the expression of several genes, including *tcdA, codY, tcdC, tcdR, luxS*, and *cwp84*, suggesting a complex regulatory network influencing these virulence factors. However, six isolates lacked the *agr2* locus, exhibiting diverse RT (four types), *tcdC* genotypes (three types), and toxinotype groups (two types). This variability may stem from divergent evolutionary trajectories within the *tcdA^+^B^+^ C. difficile* population over extended periods. A recent study by Okada *et al*. investigated the epidemiology of *agr* loci in 133 *C. difficile* isolates from Japan, revealing that 98.4 % of the isolates contained regions corresponding to *agr1*, and 65.4 % (87 isolates) were positive for the *agr2* locus. Notably, two *agr1*-negative isolates in this study were non-toxigenic and belonged to the C-I and C-III MLST-based clades. These findings suggest that *agr1* deficiency is not necessarily detrimental to bacterial survival and may occur during evolutionary processes. Additionally, an *in silico* analysis of *C. difficile* sequences deposited in GenBank indicated that all strains contained genetic regions corresponding to *agr1* of the CD630 strain. The *agr2* locus was further subcategorized into *agr2R* and *agr2M* homologs, showing approximately 80 % identity compared to the *agr2R* of the R20291 strain (Okada et al., 2020). In our study, we designed a set of specific primers to investigate the distribution and genetic diversity of *agrD1* and *agrD2* genes in clinical *C. difficile* isolates from diarrheal patients. We discovered a mutation, C140A, which substitutes threonine with lysine (T47K) in the *Agr1* peptide, present in 24 % of our isolates. This substitution showed a significant statistical correlation with the genotypes of these isolates, most of which belonged to the RT126/*tcdC-A*/toxinotype V group. According to Darkoh *et al*., the synthesis of *C. difficile* toxin is regulated by a small thiolactone molecule encoded by an accessory gene regulator system, which has been identified in the stools of CDI patients. Their research demonstrated that all sequenced *C. difficile* genomes include a cysteine-containing AgrD1 autoinducer pre-peptide (Darkoh et al., 2015). Additionally, *agr1* mutant strains are unable to generate TcdA and TcdB toxins. Despite their ability to colonize a murine CDI model, these mutants are incapable of causing disease (Darkoh et al., 2015; Darkoh, Odo, and DuPont, 2016). Another study demonstrated that *C. difficile* RT027 R20291 *agrA* mutant exhibited 75 differentially regulated transcripts during the late exponential growth phase. These transcripts included genes involved in flagellar assembly and function, c-di-GMP synthesis, and *tcdA* expression (Martin et al., 2013). Furthermore, a recent comprehensive study highlighted the presence of two principal quorum sensing (QS) systems, Agr and Lux, which play a crucial role in bacterial pathogenicity by modulating virulence factor expression, motility, and adhesion (Gunaratnam et al., 2021).

In hospitals and healthcare settings, CDI is primarily disseminated through spores. These bacterial spores are highly infectious and exhibit exceptional resistance to various harmful exposures and aerobic conditions outside the host. CDI recurrence occurs in 15 % to 35 % of patients, indicating that spore formation may play a significant role in the long-term persistence of infection despite antibiotic therapy (Phanchana et al., 2021). Our findings reveal a highly variable sporulation efficiency among clinical *tcdA^+^B^+^* isolates. Notably, isolates with the *tcdC-A* genotype exhibited a higher viability in spore formation compared to other *tcdC* genotypes. Moreover, the study revealed that *Spo0A* expression is linked to several cellular components, including *slpA, fliD, codY, cwp84*, and particularly *tcdB*. As a global transcriptional regulator, Spo0A is crucial for bacterial sporulation and has been identified as essential for disease relapse and transmission in murine models (Pettit et al., 2014). This observation aligns with previous reports that hypervirulent strains, such as RT001 and RT027, possess significant sporulation potential. The combination of enhanced sporulation traits and antibiotic resistance in these strains suggests a contributing factor to their widespread dissemination in the environment (Åkerlund et al., 2008; Fawley et al., 2007). Inactivating the *spo0A* master regulator has been shown to inhibit toxin production in *C. difficile* strains, suggesting its role as a regulator of toxin synthesis. The *spo0A* controls numerous genes during the post-exponential phase through phosphorylation and activation. Similar to our findings, Burns et al. observed significant variation in sporulation rates among different BI/NAP1/027 isolates, although this variation was not type-specific, affecting both epidemic and non-epidemic strains. They also did not find any correlation between sporulation rates and the severity of disease in BI/NAP1/027 type strains (Burns, Heap, and Minton, 2010). Another study demonstrated that toxigenic isolates exhibited differences in toxin production, sporulation rate, viable spore formation, and growth rate. However, these variations were not type-specific. Notably, isolates from patients with severe disease produced twice as many spores as those from patients with non-severe disease, suggesting that higher spore production is linked to severe CDI. Furthermore, a negative association was observed between growth rate and toxin production capacity (Carlson et al., 2013). Isolates with higher sporulation ability appear to produce higher infectious doses and transmit more easily to susceptible individuals. These isolates also have slower growth rates, allowing them to exploit their metabolic capacity for toxin production. In our study, the motility abilities of isolates varied, with no correlation to specific genotypes. *C. difficile* strains exhibit differing levels of motility in liquid or semi-solid media; for instance, RT027 and RT012 are motile strains, whereas RT078 is non-motile. An infection model in mice has shown that flagella play a critical role in bacterial colonization, with motile strains being up to ten times more likely to adhere to the cecum than non-motile strains of the same serogroup (Dingle et al., 2011). However, the sample size of 50 *tcdA+B+* isolates, while representative, may not fully encompass broader isolates diversity across different epidemiological contexts. Moreover, financial and logistical constraints have influenced the feasibility of large-scale sequencing and functional assays, which could have provided deeper mechanistic insights.

## 5 Conclusion

Our investigation has unveiled significant insights into the genetic diversity and molecular epidemiology of *agr* loci in *tcdA^+^B^+^* clinical *C. difficile* isolates from Iranian patients with CDI. The study highlights the prevalence and universality of the *agr1* locus, including the previously unrecognized T47K substitution from Iran. Additionally, we documented the distribution of two *agr* loci within *tcdA^+^B^+^* isolates. Furthermore, our research has provided detailed phenotypic characterizations of these isolates, including variations in sporulation efficiency and motility. These findings are crucial as they pave the way for future functional and clinical studies, enhancing our understanding of *C. difficile* pathogenesis and informing potential therapeutic strategies.

## Funding

The study was supported by a research grant (Project No: RIGLD 1024) from the Foodborne and Waterborne Diseases Research Center at the Research Institute for Gastroenterology and Liver Diseases, Shahid Beheshti University of Medical Sciences, Tehran, Iran.

## CRediT authorship contribution statement

**Mansoor Kodori:** Investigation, Methodology, Writing – original draft. **Zohreh Ghalavand:** Conceptualization, Supervision, Writing – review & editing. **Abbas Yadegar:** Conceptualization, Methodology, Validation, Data curation, Project administration, Writing – review & editing. **Gita Eslami:** Resources, Writing – review & editing. **Masoumeh Azimirad:** Visualization, Software, Data curation. **Mohammad Reza Zali:** Funding acquisition, Resources.

## Declaration of competing interest

The authors declare that they have no known competing financial interests or personal relationships that could have appeared to influence the work reported in this paper.

## Data Availability

Data will be made available on request.

## Appendix Supplementary materials

Image, application 1
