# Supplementary material for: In-depth characterization of accessory gene regulator loci and associated virulence factors in tcdA+B+ Clostridioides difficile isolates
Source: Curr Res Microb Sci. 2025 Jul 1;9:100435. doi: 10.1016/j.crmicr.2025.100435 (PMC12274312; doi:10.1016/j.crmicr.2025.100435)
Supplement: Supplementary file 1 [file mmc1.doc]

**Table S1. The oligonucleotide sequences used to detect *C. difficle* isolates and toxin genes in this study.**

| **Target gene** | **Primer name** | **Oligonucleotide sequence (5′–3′)** | **Product size (bp)** | **Reference** |
| --- | --- | --- | --- | --- |
| 16S rRNA | PS13  PS14 | GGAGGCAGCAGTGGGGAATA  TGACGGGCGGTGTGTACAAG | 1062 | (1) |
| *cdd3* | Tim 6  Struppi 6 | TCCAATATAATAAATTAGCATTCCA  GGCTATTACACGTAATCCAGATA | 622 | (1) |
| *cdu2* | Tim 5  Struppi 5 | CCACAGATGCTTTTAGCAGGAA  TCCAATCACTGCTCCAGCTAT | 162 | (1) |
| *tcdA* | tcdA-F3345  tcdA-R3969 | GCATGATAAGGCAACTTCAGTGGTA  AGTTCCTCCTGCTCCATCAAATG | 629 | (1) |
| *tcdB* | tcdB-F5670  tcdB-R6079A  tcdB-R6079B | CCAAARTGGAGTGTTACAAACAGGTG  GCATTTCTCCATTCTCAGCAAAGTA  GCATTTCTCCGTTTTCAGCAAAGTA | 410 | (1) |
| *tcdC* | C1  C2 | TTAAATAATTTTCTCTACAGCTATCC  TCTAATAAAAGGGAGATTGTATTATG | 718 | (1) |
| *tcdR* | Tim 3  Struppi 3 | AAAAGCGATGCTATTATAGTCAAA  CCTTATTAACAGCTTGTCTAGAT | 300 | (1) |
| *tcdE* | Tim 1  Struppi 1 | GTTTAAGTGCAATAAAAAGTCGTA  GGTAATCCACATAAGCACATATT | 262 | (1) |
| *cdtA* | cdtA-F739A  cdtA-F739B  cdtA-R958 | GGGAAGCACTATATTAAAGCAGAAGC  GGGAAACATTATATTAAAGCAGAAGC  CTGGGTTAGGATTATTTACTGGACCA | 221 | (1) |
| *cdtB* | ctdB-F617  cdtB-R878 | TTGACCCAAAGTTGATGTCTGATTG  CGGATCTCTTGCTTCAGTCTTTATAG | 262 | (1) |

**Table S2.** Primers sets of *agr* loci and virulence factors used in this study.

| **Target gene** | **Oligonucleotide sequences (5′-3′)** | **Product size (bp)** | **Assay** | **Reference** |
| --- | --- | --- | --- | --- |
| *agrD1* | F: ATGTTTTGGATTTTTGTGATGTTAG  R: CCAAATAGGTATTAGGCTCC | 359 | PCR, qPCR | This study |
| *agrD2* | F: GTGGTGAAACAATAGTTAATTAC  R: ATAATCTTTTAAACATATTTCCTCC | 297 | PCR, qPCR | This study |
| *agrB1* | F: ATATGCTGAAAAAATGACATCTG  R: CAAAGAAAGACTTAAAATTGT | 475 | PCR, qPCR | This study |
| *agrB2* | F: TAATGAGATTGTAGAAAGTGAAG  R: ACGAATAGTATTTTTTGCACAG | 408 | PCR, qPCR | This study |
| *agrA2* | F: TTTAAGAATTGTGATTAGTATAGG  R: TTCTATTTTAGATATACTCATTTTAG | 538 | PCR, qPCR | This study |
| *agrC2* | F: TAGTTGGAAAGAGGAAAATATC  R: TATTTTAATCTATCATGAACTTCG | 670 | PCR, qPCR | This study |
| *codY* | F: TTACAAACAAGTGGTGGAAGCAG  R: CCTATTGCCATTTGAACTACAGC | 488 | PCR | This study |
| *codY* | F: GGAAGCGGTCAAAGATTAGG  R: GCTTCAAGCTCGGAGTAGGA | 206 | qPCR | This study |
| *fbp68* | F: AATTATCATCAAAACTTACAGGTG  R: AACTTGACGAACTCTACTTATGC | 431 | PCR | This study |
| *fbp68* | F: AGTTCGTCAAGTTTTACCTGGTC  R: GGTCCTTCCAATTCCTCTAGGT | 120 | qPCR | (2) |
| *luxS* | F: ATGTAGAAAGTTTTGGAACTGCTG  R: GTTTTGGCATCTACATCTCCC | 154 | qPCR | This study |
| *fliD* | F: ACTAAATCTGCAGTAGTATATG  R: TTATTTTGATTTTTTAGTAGTAAC | 261 | PCR | (3) |
| *slpA* | F: GTTGGGAGGAATTTAAGRAATG  R: GCWGTYTCTATTCTATCDTYWCC* | 1200 | PCR | (4) |
| *cwp84* | F: TGGGCAACTGGTGGAAAATA  R: TAGTTGCACCTTGTGCCTCA | 151 | PCR, qPCR | (5) |
| *fliD* | F: AACTGGTTCGTCATCAGACATT  R: TACACTTGCCACTTTGTTTCCA | 124 | qPCR | (3) |
| *slpA* | F: AATGATAAAGCATTTGTAGTTGGTG  R: TATTGGAGTAGCATCTCCATC | 126 | qPCR | (2) |
| *spo0A* | F: AGCGCAATAAATCTAGGAGCA  R: TGGCTCAACTTGTGTAACTCTAT | 111 | qPCR | (6) |
| *ccpA* | F: GTGATGAGGCTGCCATAGGT  R: TTGCTACTGCTCCCATATCGT | 165 | qPCR | (7) |
| *tcdA* | F: TCTACCACTGAAGCATTAC  R: TAGGTACTGTAGGTTTATTG | 157 | qPCR | (8) |
| *tcdB* | F: ATATCAGAGACTGATGAG  R: TAGCATATTCAGAGAATATTG | 103 | qPCR | (8) |
| *tcdC* | F: TGGCATTTATTTTAGGCGTGT  R: GCTTTCTTTTCGTCGTCTTTC | 183 | qPCR | (9) |
| *tcdR* | F: GACTTTTGAGTTGTCTA  R: CTTTTCTTTGAATACCAG | 167 | qPCR | (9) |
| *cdtA* | F: GGGAAGCACTATATTAAAGCAGAAGC  R: CTGGGTTAGGATTATTTACTGGACCA | 220 | qPCR | (10) |
| *cdtB* | F: TTGACCCAAAGTTGATGTCTGATTG  R: CGGATCTCTTGCTTCAGTCTTTATAG | 260 | qPCR | (10) |
| *rpoA* | F: TCATTACCAGGTGTAGCAGTGAA  R: GAGCATGGTCCTTGAGCTTC | 179 | qPCR | (11) |

W = A or T, Y =C or T, R= A or G, D=A or G or T.

**Table S3.** Demographic and clinical data of CDI patients included in this study.

| **Characteristics** | | **Number of patients (%) (N=50)** |
| --- | --- | --- |
| Gender | Male | 21 (42)  29 (58) |
|  | Female |  |
| Hospital ward | Internal medicine | 5 (10)  3 (6)  2 (4)  3 (6)  8 (16)  2 (4)  19 (38)  1 (2)  3 (6)  1 (2)  1 (2)  2 ($) |
|  | Infectious |  |
|  | Surgery |  |
|  | ICU |  |
|  | Oncology |  |
|  | CCU |  |
|  | Gastroenterology |  |
|  | Endocrinology |  |
|  | Nephrology |  |
|  | Orthopedics |  |
|  | Psychology |  |
|  | Out-patients |  |
| Hospitalization (in the last three months) | Yes | 8 (16)  42 (84) |
|  | No |  |
| Defecation (times/day) | 3-5 | 27 (54)  12 (24)  3 (6)  8 (16) |
|  | 5-8 |  |
|  | 8-10 |  |
|  | >10 |  |
| Drug consumption | Immunosuppressive | 11 (22)  10 (2)  39 (78) |
|  | Antacid |  |
|  | Antibiotic |  |

ICU, intensive care unit; CCU, coronary care unit.

**Table S4**. Typing of isolates according to *tcdC* gene sequence, RT, and toxinotyping methods.

| **RT (N)** | ***tcdC* genotype (N)** | **Toxinotype (N)** | ***agr* type (N)** | ***cdt*** |
| --- | --- | --- | --- | --- |
| 001 (13) | wild-type (5)  tcdC-sc3 (6)  tcdC-A (1)  tcdC-14 (1) | 0 (5)  0 (5), XIII (1)  0 (1)  0 (1) | *agr1* and *agr2* (3), *agr1* (2)  *agr1* and *agr2* (6)  *agr1* (1)  *agr1* (1) | Negative |
| 126 (10) | tcdC-A (9)  tcdC-sc3 (1) | V (8), XXVIII (1)  V (1) | *agr1* and *agr2* (10) | Positive |
| 014 (3) | tcdC-sc9 (2)  tcdC-A (1) | 0 (3) | *agr1* and *agr2* (1), agr1 (1)  *agr1* and *agr2* (1) | Negative |
| 005 (3) | wild-type (2)  tcdC-sc9 (1) | 0 (2)  XIII (1) | *agr1* and *agr2* (3) | Negative  Positive |
| 070 (3) | tcdC-sc9 (3) | 0 (2)  XIII (1) | *agr1* and *agr2* (2)  *agr1* (1) | Negative |
| 038 (2) | tcdC-sc3 (1)  wild-type (1) | 0/V (1)  V (1) | *agr1* and *agr2* (2) | Positive |
| 029 (2) | wild-type (2) | 0 (2) | *agr1* and *agr2* (2) | Negative |
| 103 (1) | tcdC-sc3 (1) | 0 (1) | *agr1* and *agr2* (1) | Negative |
| 085 (1) | tcdC-sc3 (1) | 0 (1) | *agr1* and *agr2* (1) | Positive |
| WRT628 (1) | wild-type (1) | V (1) | *agr1* and *agr2* (1) | Negative |
| 039 (1) | tcdC-sc15 (1) | 0 (1) | *agr1* and *agr2* (1) | Negative |
| 015 (1) | wild-type (1) | 0 (1) | *agr1* and *agr2* (1) | Negative |
| 019 (1) | tcdC-B (1) | 0 (1) | *agr1* and *agr2* (1) | Negative |
| 004 (1) | tcdC-sc9 (1) | 0 (1) | *agr1* and *agr2* (1) | Negative |
| 003 (2) | wild-type (2) | 0 (2) | *agr1* and *agr2* (2) | Negative |
| 405 (1) | tcdC-sc9 (1) | XIII (1) | *agr1* and *agr2* (1) | Negative |
| 139 (1) | tcdC-sc9 (1) | 0 (1) | *agr1* and *agr2* (1) | Negative |
| Unrecognized (3) | tcdC-sc3 (2)  wild-type (1) | 0 (3) | *agr1* and *agr2* (3) | Negative |

RT, ribotype.

**Table S5.** Fifteen reference strains were obtained from GenBank for comparative genomic analysis in this study.

| **Strain ID** | **GenBank accession no.** | **RT** | **Source** | **Year** |
| --- | --- | --- | --- | --- |
| CD21062 | CP033216 | 78 | China | 2015 |
| R20291 | FN545816 | 27 | UK | 2006 |
| 2007855 | FN665654 | 27 | US | 2007 |
| 08ACD0030 | CP010888 | - | - | - |
| CD630 | CP010905 | 12 | Switzerland | 1982 |
| CF5 | FN665652 | 17 | Belgium | 1995 |
| M120 | NC_017174 | 78 | UK | 2007 |
| M68 | FN668375.1 | 17 | Ireland | 2006 |
| DSM1296 | CP011968.1 | - | Germany | 2015 |
| DSM29629 | CP016104.1 | SLO 235 | Indonesia | 2014 |
| CD161 | CP029154.1 | - | China | 2013 |
| DSM29627 | CP016102.1 | 17 | Indonesia | 2014 |
| DSM28669 | CP012323.1 | SLO 091 | Ghana | 2016 |
| CBA7204 | CP029566.1 | 18 | South Korea | 2017 |
| CDT4 | CP029152.1 | - | China | 2014 |

RT, ribotype.





**Figure S1**. Comparative basal expression levels of pathogenicity locus (PaLoc) genes normalized to the housekeeping *rpoA* gene across all isolates**.** A: *tcdA*; B: *tcdB*; C: *tcdC*; D: *tcdR*.





**Figure S2**. Comparative basal expression levels of quorum sensing genes normalized to the housekeeping *rpoA* gene across all isolates**.** A: *luxS*; B: *agrD1*; C: *agrD2*.





**Figure S3**. Comparative basal expression levels of cell surface-associated genes normalized to the housekeeping *rpoA* gene across all isolates. A: *fbp68*; B: *slpA*; C: *cwp84*; D: *fliD*.





**Figure S4.** Comparative basal expression levels of key regulatory genes normalized to the housekeeping *rpoA* gene across all isolates. A: *spo0A*; B: *ccpA*; C: *codY*.

**References**

1. Kodori M, Ghalavand Z, Yadegar A, Eslami G, Azimirad M, Krutova M, et al. Molecular characterization of pathogenicity locus (PaLoc) and tcdC genetic diversity among tcdA+ B+ Clostridioides difficile clinical isolates in Tehran, Iran. 2020;66:102294.

2. Denève C, Bouttier S, Dupuy B, Barbut F, Collignon A, Janoir CJAa, et al. Effects of subinhibitory concentrations of antibiotics on colonization factor expression by moxifloxacin-susceptible and moxifloxacin-resistant Clostridium difficile strains. 2009;53(12):5155-62.

3. Negri A, Potocki W, Iwanicki A, Obuchowski M, Hinc KJJomm. Expression and display of Clostridium difficile protein FliD on the surface of Bacillus subtilis spores. 2013;62(9):1379-85.

4. Kato H, Kato H, Ito Y, Akahane T, Izumida S, Yokoyama T, et al. Typing of Clostridium difficile isolates endemic in Japan by sequencing of slpA and its application to direct typing. 2010;59(5):556-62.

5. Hamada M, Yamaguchi T, Ishii Y, Chono K, Tateda KJJoI, Chemotherapy. Inhibitory effect of fidaxomicin on biofilm formation in Clostridioides difficile. 2020;26(7):685-92.

6. Chen K-Y, Rathod J, Chiu Y-C, Chen J-W, Tsai P-J, Huang IJFic, et al. The transcriptional regulator Lrp contributes to toxin expression, sporulation, and swimming motility in Clostridium difficile. 2019;9:356.

7. Girinathan BP, Ou J, Dupuy B, Govind RJPp. Pleiotropic roles of Clostridium difficile sin locus. 2018;14(3):e1006940.

8. Zhu S, Zhang H, Zhang X, Wang C, Fan G, Zhang W, et al. Investigation of toxin gene diversity and antimicrobial resistance of Clostridium difficile strains. 2014;2(5):743-8.

9. Lee ASY, Song KPJB, communications br. LuxS/autoinducer-2 quorum sensing molecule regulates transcriptional virulence gene expression in Clostridium difficile. 2005;335(3):659-66.

10. Persson S, Torpdahl M, Olsen KJCm, infection. New multiplex PCR method for the detection of Clostridium difficile toxin A (tcdA) and toxin B (tcdB) and the binary toxin (cdtA/cdtB) genes applied to a Danish strain collection. 2008;14(11):1057-64.

11. Merrigan M, Venugopal A, Mallozzi M, Roxas B, Viswanathan V, Johnson S, et al. Human hypervirulent Clostridium difficile strains exhibit increased sporulation as well as robust toxin production. 2010;192(19):4904-11.
